# Preclinical immunotherapy with Cytokine-Induced Killer lymphocytes against epithelial ovarian cancer

**DOI:** 10.1038/s41598-020-63634-z

**Published:** 2020-04-15

**Authors:** S. Capellero, J. Erriquez, C. Melano, G. Mesiano, S. Genta, A. Pisacane, G. Mittica, E. Ghisoni, M. Olivero, M. F. Di Renzo, M. Aglietta, D. Sangiolo, G. Valabrega

**Affiliations:** 1 Department of Oncology, University of Torino, Torino, Italy; 2 Candiolo Cancer Institute, FPO-IRCCS, Candiolo, TO Italy; 3 Unit of Oncology, ASL Verbano Cusio Ossola (VCO), Verbania, Italy

**Keywords:** Ovarian cancer, Tumour immunology

## Abstract

Despite improvements in surgery and medical treatments, epithelial ovarian cancer (EOC) remains the most lethal gynaecological malignancy. Aim of this study is to investigate the preclinical immunotherapy activity of cytokine-induced killer lymphocytes (CIK) against epithelial ovarian cancers, focusing on platinum-resistant settings. We generated CIK *ex vivo* starting from human peripheral blood samples (PBMCs) collected from EOC patients. Their antitumor activity was tested *in vitro* and *in vivo* against platinum-resistant patient-derived ovarian cancer cells (pdOVCs) and a Patient Derived Xenograft (PDX), respectively. CIK were efficiently generated (48 fold median *ex vivo* expansion) from EOC patients; pdOVCs lines (n = 9) were successfully generated from metastatic ascites; the expression of CIK target molecules by pdOVC confirmed pre and post treatment *in vitro* with carboplatin. The results indicate that patient-derived CIK effectively killed autologous pdOVCs *in vitro*. Such intense activity was maintained against a subset of pdOVC that survived *in vitro* treatment with carboplatin. Moreover, CIK antitumor activity and tumor homing was confirmed *in vivo* within an EOC PDX model. Our preliminary data suggest that CIK are active in platinum resistant ovarian cancer models and should be therefore further investigated as a new therapeutic option in this extremely challenging setting.

## Introduction

Standard therapy of epithelial ovarian cancer (EOC) includes cytoreductive surgery to no residual disease associated with platinum-based chemotherapy^1–3^. Unfortunately recurrence rate of remains high and at the day EOC represent the main cause of death among gynecological neoplasm for women in developed countries^4,5^. It’s reported that platinum-based re-challenging brings benefit in platinum-sensitive patients, while platinum-resistant patients are treated with single-agent chemotherapy (e.g., pegylated liposomal doxorubicin^6,7^ paclitaxel^8^ gemcitabine^9^, topotecan^10,11^, or docetaxel^11,12^). Several new drugs have been recently approved, such as bevacizumab^13–16^ and PARP inhibitors^17–23^, for BRCA-mutated EOCs, but nevertheless the prognosis remains severe and it is necessary discovered new strategies to prevent tumor spreading, progression and overcome drug resistance.

In the last few years strong evidence showed that immunotherapy is effective both in solid and hematological malignancies. Currently, most of the efforts are directed towards immune checkpoint inhibitors (anti PD-1, PD-L1, CTLA4) which are able to induce long lasting remissions in specific settings of metastatic solid tumors (e.g. melanoma, Non small cell lung cancer), although if still in limited numbers of patients^24–27^. Recently, immune checkpoint inhibitors have shown activity but only in small subsets of EOC patients with BRCA mutations or microsatellite instability (MSI)^28^. Defects in tumor antigen presentation and antigen-presenting machinery are emerging among the possible causes of resistance to immunotherapy with checkpoint inhibitors^29^. An MHC unrestricted cell based approach with cytokine-induced killer (CIK) lymphocytes could help to overcome some limitations emerged with other immunotherapies based on checkpoint inhibitors^30–34^. CIK, firstly described in the early 1990s, exert MHC-unrestricted antitumor activity^35,36^ and represent a subgroup of heterogeneous T lymphocytes expanded *ex vivo* with mixed T-NK phenotype. CIK can be easily expanded starting from peripheral blood mononuclear cells (PBMC), cord blood^37,38^, bone marrow^39^ or other sources^40^, in presence of INF-ϒ, Ab-anti-CD3 and interleukin 2 (IL2)^41^. The cytotoxic activity is mostly mediated by the interaction of their NKG2D membrane receptor with several members of stress-inducible molecules expressed on tumors, such as UL-16–binding proteins (ULBPs) and MHC class I-related chain A and B (MIC A/B)^42,43^.

It has already been reported that MICA/B and ULBPs are expressed on EOC tumors and are associated with poor prognosis^44,45^.

Strong preclinical evidence^46–49^ and early clinical trials with CIK have shown encouraging findings in challenging settings such as metastatic lung cancer, liver cancer, cervical cancer, gastrointestinal cancer, leukemia, soft tissue-sarcoma and melanoma. Moreover, some preclinical works underscore the killing capacity of CIK even against ovarian cancer cells *in vitro*^30,50,51^. We built on this evidence and focused our attention on the setting of platinum resistant ovarian cancer, exploring the antitumor activity of autologous CIK against chemoresistance patient-derived ovarian cancer cell lines (pdOVC).

## Results

### Generation and characterization of patient-derived ovarian cancer cell lines (pdOVC)

pdOVC were successfully established from patients with advanced EOC in 4 to 12 weeks; characteristics of the 9 patients are shown in Table 1. Notably, 6 out of 9 pdOVC cultures were established from relapsed heavily treated chemotherapy resistant patients, while the remaining 3 were established from first relapse.

**Table 1:** Main characteristics of ovarian cancer patients and corresponding tumor cell lines.

ID	Age	Mutated germline BRCA	Stage at diagnosis	Histology	Tumor grade	Number of previous lines of chemotherapy	Chemotherapic agents employed
pdOVC 02	73	BRCA 1 mutated	IV	serous	G3	2	carboplatin, paclitaxel
pdOVC 03	69	unknown	IV	serous	G3	2	cisplatin, PLD
pdOVC 04	61	unknown	IIIc	serous	G3	2	carboplatin, paclitaxel
pdOVC 05	45	unknown	IIIc	serous	G3	3	carboplatin, paclitaxel, bevacizumab, trabectedin, gemcitabine
pdOVC 06	53	unknown	IIIc	serous	G1	1	carboplatin, paclitaxel
pdOVC 07	56	BRCA 2 mutated	IIIc	serous	G3	0	na
pdOVC 08	67	unknown	unknown	serous	borderline	2	carboplatin, paclitaxel
pdOVC 12	43	BRCA 1 mutated	IIIc	serous	G3	0	na
pdOVC 14	52	unknown	IV	serous	G3	0	na

Abbreviations: na: not applicable.

**Figure 1:**
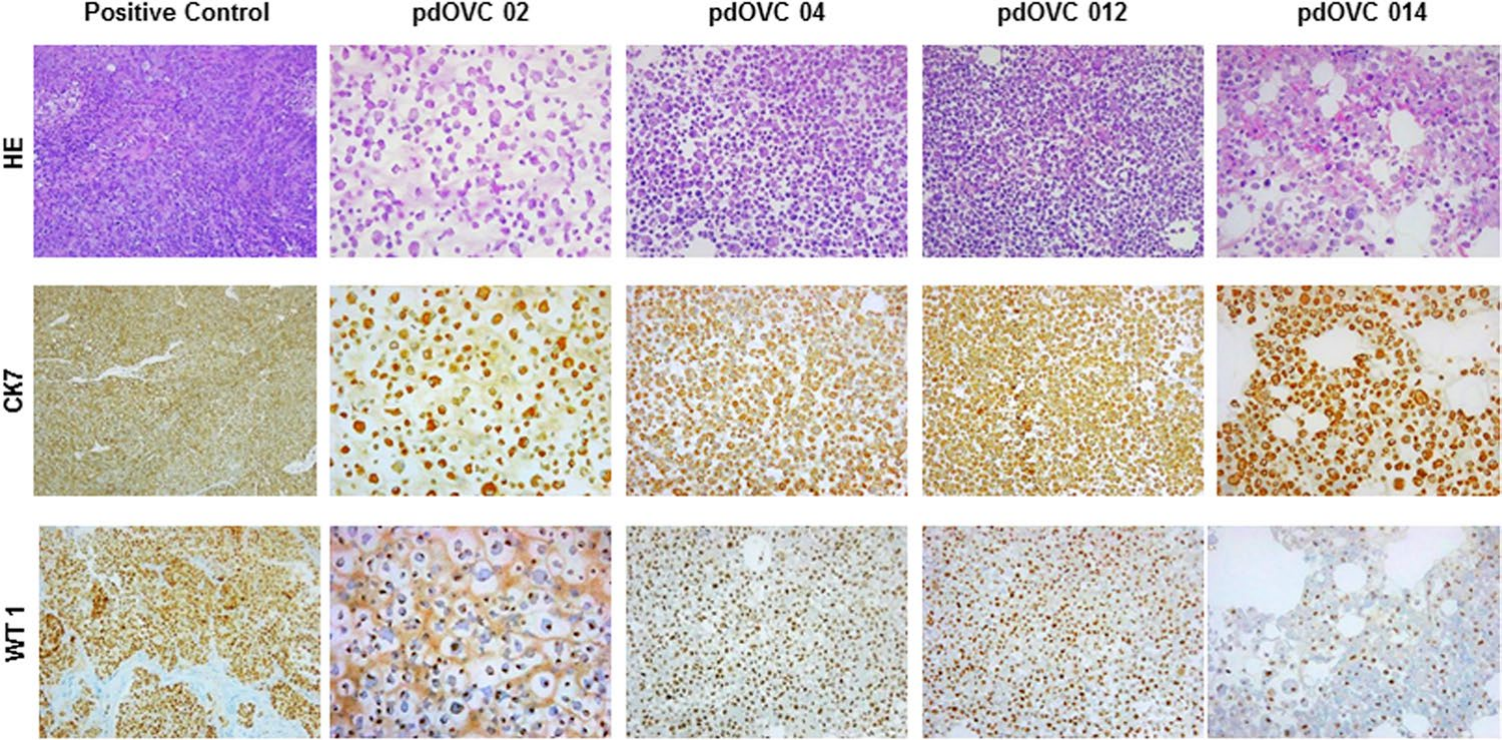
Patient derived ovarian cancer cell lines retain the expression of ovarian cancer marker WT1 and cytokeratin 7. Images of pOVC cell cultures obtained from ascites of patients affected by ovarian cancer. Image shows representative pictures of four different cultures expressing ovarian cancer marker WT1 and epithelial marker CK7 (10X magnitude). Positive control: high grade serous epithelial ovarian cancer; Abbreviations: WT1: Wilms’ Tumor 1; CK7: cytokeratin 7; HE: hematoxiliyn eosin.

Patient-derived ovarian cancer cells displayed morphologic features consistent with the pathology evaluation of the corresponding tumor and were characterized with IHC analysis for the expression of ovarian cancer marker WT1^52^ and epithelial marker cytokeratin 7^53^ (Fig. 1). We explored the expression of MHC class I molecules on all pdOVC (Table 2), along with their membrane expression of main ligands recognized by CIK (MICA/B, ULBP1, ULBP2/5/6, ULBP3). We found that pdOVC presented variable but consistent expression of ULBP2/5/6, (median 95%,range 22–99%), MICA/B (median 37,5%, range 10%-93. All pdOVCs expressed HLA class I molecules (median 94%, range 72–99%); comparable expression of CIK ligands was confirmed in 5 commercially available ovarian cancer cell lines: A2780, IGROV-1, OAW42, OVCAR3, OVCAR 5 (Table 2).

**Table 2:** Membrane expression of NKG2D ligands and HLA class I molecules by ovarian cancer cell lines.

ID	MIC A/B*	ULBP2,5,6*	Class-I HLA*
pdOVC 02	ne	63	97
pdOVC 03	ne	99	93
pdOVC 04	91	99	99
pdOVC 05	ne	22	90
pdOVC 06	26	68	94
pdOVC 07	93	99	72
pdOVC 08	49	93	96
pdOVC 09	10	97	94
pdOVC 12	18	95	91
MEDIAN	37,5	61	94
OVCAR 3	59	91	84
OVCAR 5	11	92	98
OAW42	48	53	97
A2780	9	24	96
IGROV 1	ne	48	94
MEDIAN	29,5	53	96

*Values are expressed as percentage of viable positive cells assessed by flow cytometry.

Abbreviations: ne: not expressed.

### CIK efficiently kill ovarian cancer *in vitro*

**Figure 2:**
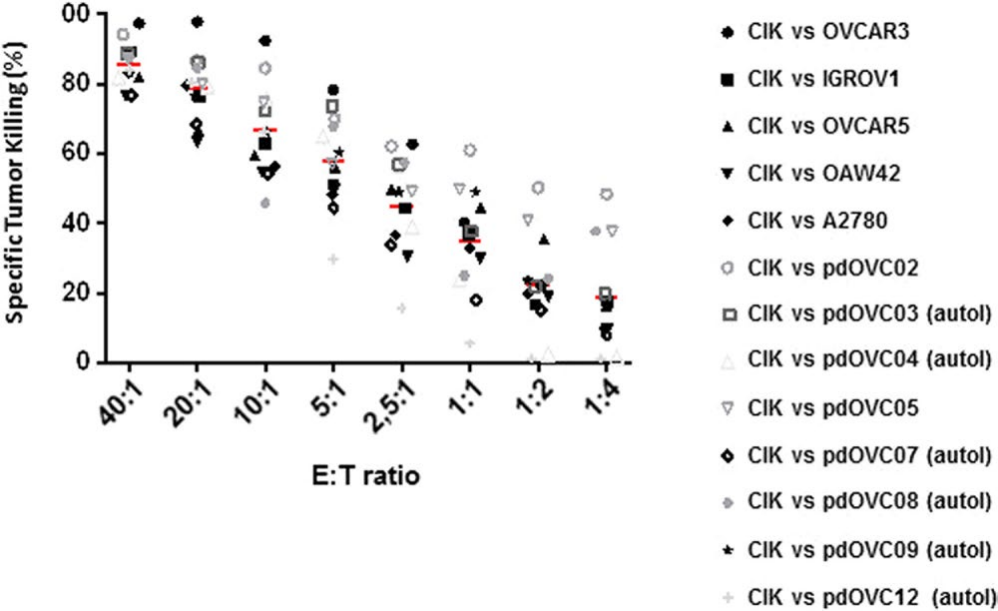
Patient-derived CIK effectively kill ovarian cancer cells. CIK lymphocytes efficiently killed *in vitro* ovarian cancer targets, including 6 cell lines generated from metastatic ascites post failure of platinum chemotherapy. CIK were autologous in 6/13 experiments. Tumor killing was assessed by CellTiter-Glo Luminescent Cell Viability Assay following 72 hour co-culture of mature CIK with ovarian targets. Symbols represent the average mortality for each pdOVC (n = 3 for each target), red dash represents mean values of tumor-specific killing for each E/T ratio.

We *ex vivo* expanded CIK from 14 patients suffering from EOC; CIK were obtained starting from fresh PBMCs cultured with the timely addition of IFN-γ, Ab-anti-CD3, and IL-2. Median expansion of bulk CIK, after 3–4 weeks of culture, was 48 fold (range 12–88). The median rate of mature CIK co-expressing CD3 and CD56 molecules (CD3^+^CD56^+^) was 33% (range 19–61%), and 87% (range 73–96%) of CIK were also CD8^+^. The median membrane expression of the NKG2D receptor, which is the main receptor responsible for tumor recognition, was 90% (range: 78–97%). A summary of patient characteristics and the relevant CIK expansion data are reported in Table 3. In selected experiments we performed a deeper phenotype analysis, including the additional i) antitumor receptor DNAM (median expression 90, range 85–99), ii) immune-checkpoint: molecules PD1 (median expression 31, range 10–60), TIM3 (median expression 64, range 41–93), LAG3 (median expression 6, range 0–15), TIGIT (median expression 29, range 17–35), iii) Natural Killer activation molecules: NKp44 (median expression 1, range 2–1), NKp 30 (median expression 9, range 8–13), NKp46 (median expression 2, range 1–6), iv) TCRαβ (median expression 96, range 87–97), TCRϒδ (median expression 2, range 1–9), v) lymphocyte subsets: effector memory (EM, median expression 63, range 42–65), effector memory-RA (EM RA median expression 20, range 13–30), central memory (CM, median expression 8, range 6–9), Naive (median expression 15, range 12–17) (Supplementary Fig. 1). At the end of CIK expansion we tested their capability to kill ovarian cancers *in vitro*. We could reproduce the autologous CIK/pdOVC setting in 6 experiments, while allogenic targets (2 pdOVC and 5 commercially available ovarian cancer cell lines) were used in 7 cases when autologous PBMC were not available. Mean values of tumor specific killing at decreasing effector/target (E/T) ratios were 82% ± 3 (40:1), 73% ± 2 (20:1), 61% ± 3 (10:1), 49% ± 3(5:1), 34% ± 6 (2,5:1), 26% ± 5 (1:1), 15% ± 4 (1:2), 12% ± 3 (1:4), assessed by luminescent cell viability assays (Fig. 2). The killer activity of CIK was further confirmed by flow cytometry based independent experiments. We did not observe any difference in the intensity of CIK tumor killing against either autologous or allogeneic pdOVC targets (n = 6) (Supplementary Fig. 2).

**Table 3:** Patient characteristics and corresponding generation of CIK cells.

ID	Age	Mutated germline BRCA	Stage at diagnosis	Histology	Tumor grade	Number of previous lines of chemotherapy	Chemotherapic agents employed		Mature NKGD2D°	Mature CD3/CD8°	Mature CD3/CD56°	Final CD3 Fold Increase
CIK 01	78	BRCA 1 mutated	IV	serous	G2	1	carboplatin, paclitaxel		90	96	46	70
CIK 02	73	BRCA 1 mutated	IV	serous	G3	2	carboplatin, paclitaxel		93	90	34	34
CIK 03*	69	unknow	IV	serous	G3	2	cisplatin, PLD		97	87	21	12
CIK 04*	61	unknow	IIIc	serous	G3	2	carboplatin, paclitaxel		78	90	37	40
CIK 05	45	unknow	IIIc	serous	G3	3	carboplatin, paclitaxel, bevacizumab, trabectedin, gemcitabine		87	73	30	18
CIK 06*	53	unknow	IIIc	serous	G1	1	carboplatin, paclitaxel		96	94	27	87
CIK 07*	56	BRCA 2 mutated	IIIc	serous	G3	0	na		89	90	36	77
CIK 08*	67	unknow	unknown	serous	borderline	2	carboplatin, paclitaxel		94	79	40	88
CIK 09	56	unknow	unknown	serous	G3	2	carboplatin, paclitaxel		86	91	61	55
CIK 10	45	unknow	IIIc	serous	G3	1	carboplatin, paclitaxel, bevacizumab		93	94	19	15
CIK 11	59	unknow	IIIc	serous	G3	1	carboplatin, paclitaxel		90	87	27	39
CIK 012*	43	BRCA 1 mutated	IIIc	serous	G3	0	Na		87	94	33	36
CIK 13	58	WT	IV	na	na	1	PLD		74	75	44	53
CIK 14	71	WT	IIIC	serous	G3	2	PLD		76	73	20	83
								MEDIAN	90	87	34	48

*Patients from whom we generated pdOVC cell lines.

°Value expressed as percentage of viable positive cells.

Abbreviations: na: not applicable.

**Figure 3:**
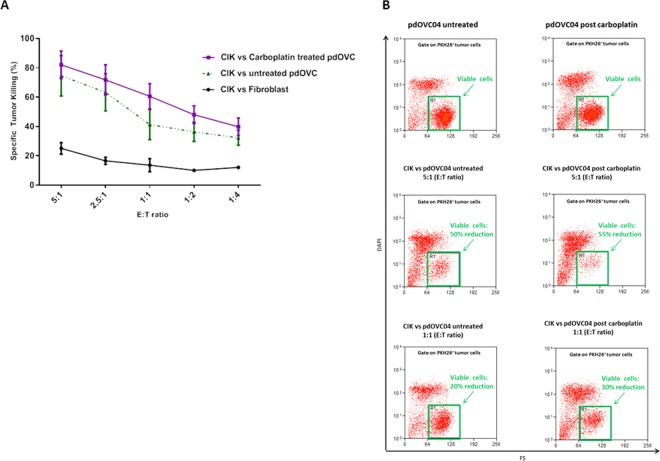
*In vitro* activity of CIK against chemo-surviving pdOVC. (**A**) CIK are capable of killing residual pdOVC that survived therapeutic doses (IC_50_, 72 hours) of carboplatin *in vitro*. CIK cytotoxicity is fully comparable, with a trend to superiority, with paired control against untreated pdOVC. (**B)** Tumor killing was evaluated by flow cytometry. The figure shows representative flow-cytometry dot plots reporting the decrease of viable tumor cells during treatment with CIK.

**Figure 4:**
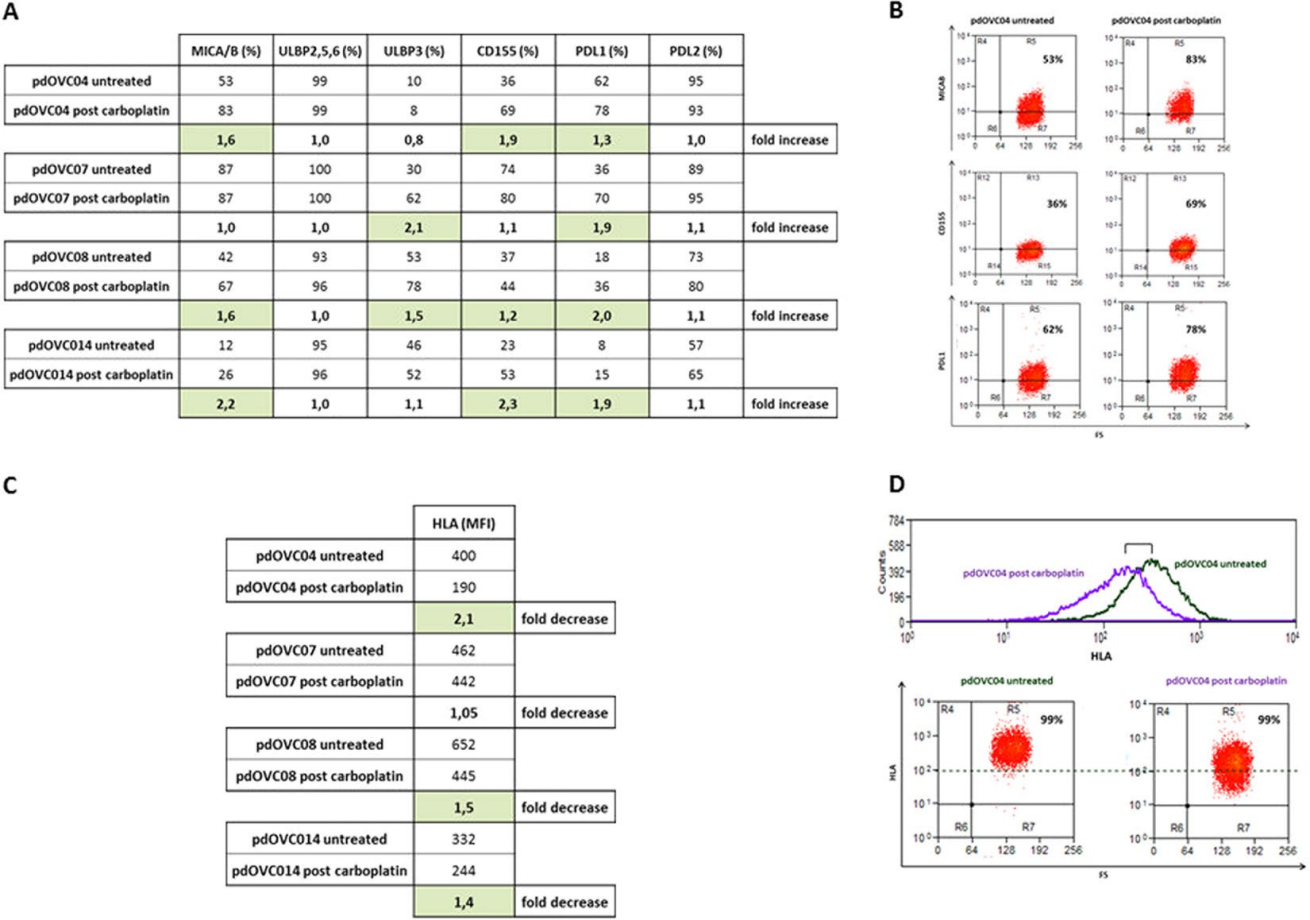
Membrane expression of NKG2D ligands in pdOVC after treatment with carboplatin. **(A,B)** The expression by pdOVC of target molecules recognized by CIK, NKG2D and DNAM ligands were confirmed to be highly expressed, even trending to increase, after *in vitro* treatment with Carboplatin, sustaining the observed effective cytotoxic effect. Expression rates for each molecule and representative flow cytometry dot plots are reported. (**C)** A relative trend toward decreased expression of HLA class I molecules was observed in pdOVC surviving chemotherapy treatment *in vitro*. Representative flow-cytometry histograms and plots are reported.

In selected experiments (n = 4) we explored and confirmed that patient-derived CIK effectively kill pdOVC that survived a previous treatment *in vitro* with therapeutic doses (30 μM, IC_50_) of Carboplatin. The killing activity was comparable, with a clear trend toward superiority, to that observed versus paired platinum-untreated controls. The mean values of tumor specific killing for platinum-surviving pdOVC, and respective platinum-untreated controls, were: 82% vs 74% (E/T 5:1), 72% vs 63% (E/T 2,5:1), 60% vs 41% (E/T 1:1), 48% vs 36% (E/T 1:2), 39% vs 32% (E/T 1:4) (Fig. 3A,B). We observed that stress-inducible NKG2D ligands, recognized by CIK, trended to be higher on pdOVC that survived the *in vitro* treatment with carboplatin: mean values expression were 48,5% vs 65,75% for MICAB, 39,75% vs 50% for ULBP3, 42,5% vs 61,5% for CD155, 31% vs 49,75% for PDL1, comparing untreated pdOVC with platinum-surviving pdOVC (Fig. 4A,B). These results support the rationale for the observed enhanced killing by CIK (Fig. 3A,B). The role of the NKG2D receptor was further confirmed in selected experiments where its selective blocking sensibly impaired reduction, 69% vs 38% (E/T 5:1) and 59% vs 33% (E/T 1:1), even if not abrogated, the pdOVC killing by CIK (Supplementary Figure 3).

Interestingly, following the treatment *in vitro* with carboplatin, the residual pdOVC presented a lower intensity of HLA-I expression (Fig. 4C,D), providing further relevance to the potential benefit in clinical perspective from an HLA-independent approach with CIK immunotherapy.

### *In vivo* activity of CIK against ovarian cancer

**Figure 5:**
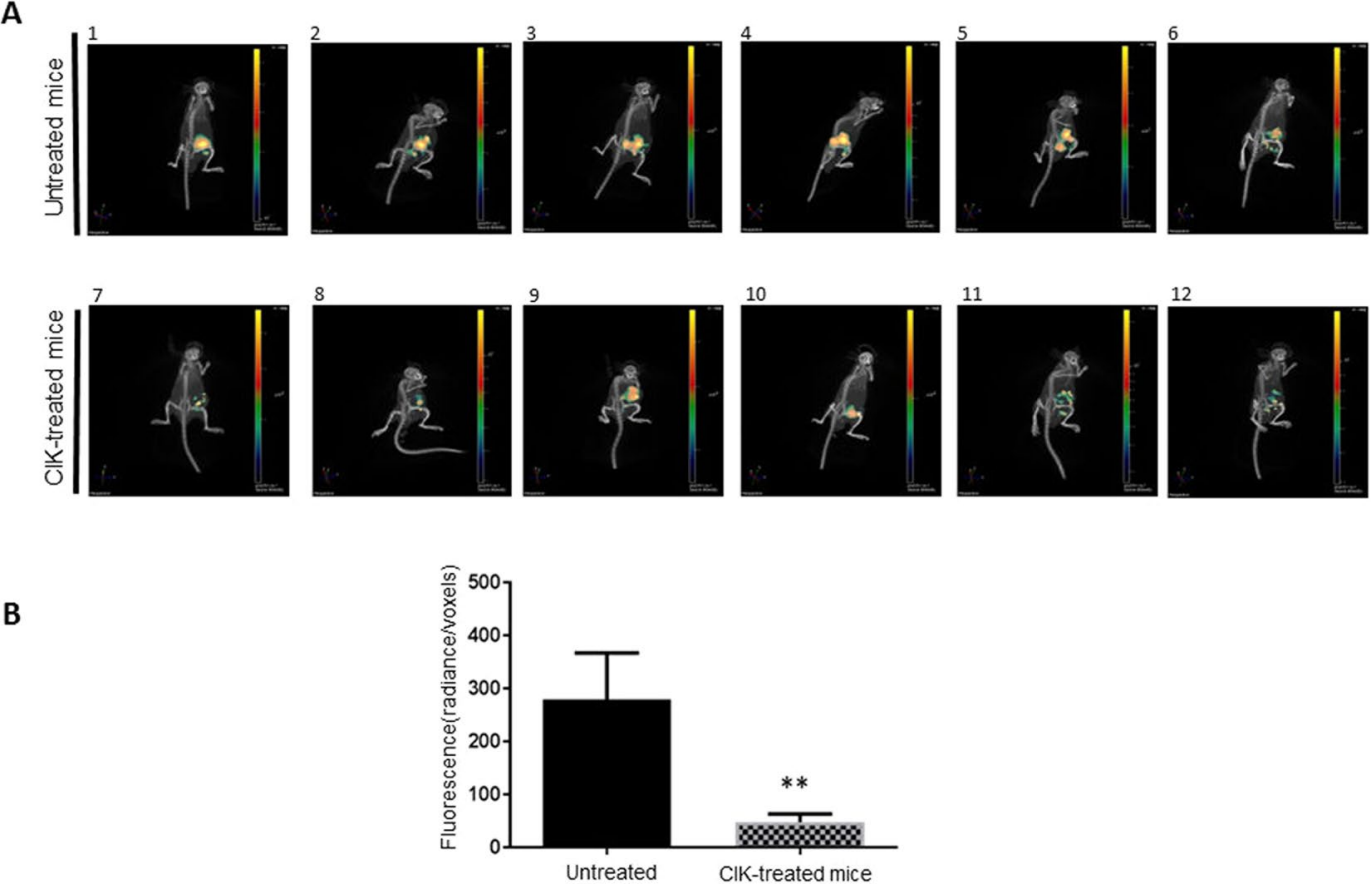
CIK activity in ovarian cancer PDX. (**A,B)** The antitumor activity of patient-derived CIK was confirmed *in* vivo within a patient-derived high grade serous (chemo-naïve) ovarian cancer PDX model. The antitumor activity was evaluated by measuring variations in the tumor-glucose uptake *in vivo*. Representative pictures of tumor-glucose uptake for each mice and cumulative analysis are reported and compared with untreated controls.

**Figure 6:**
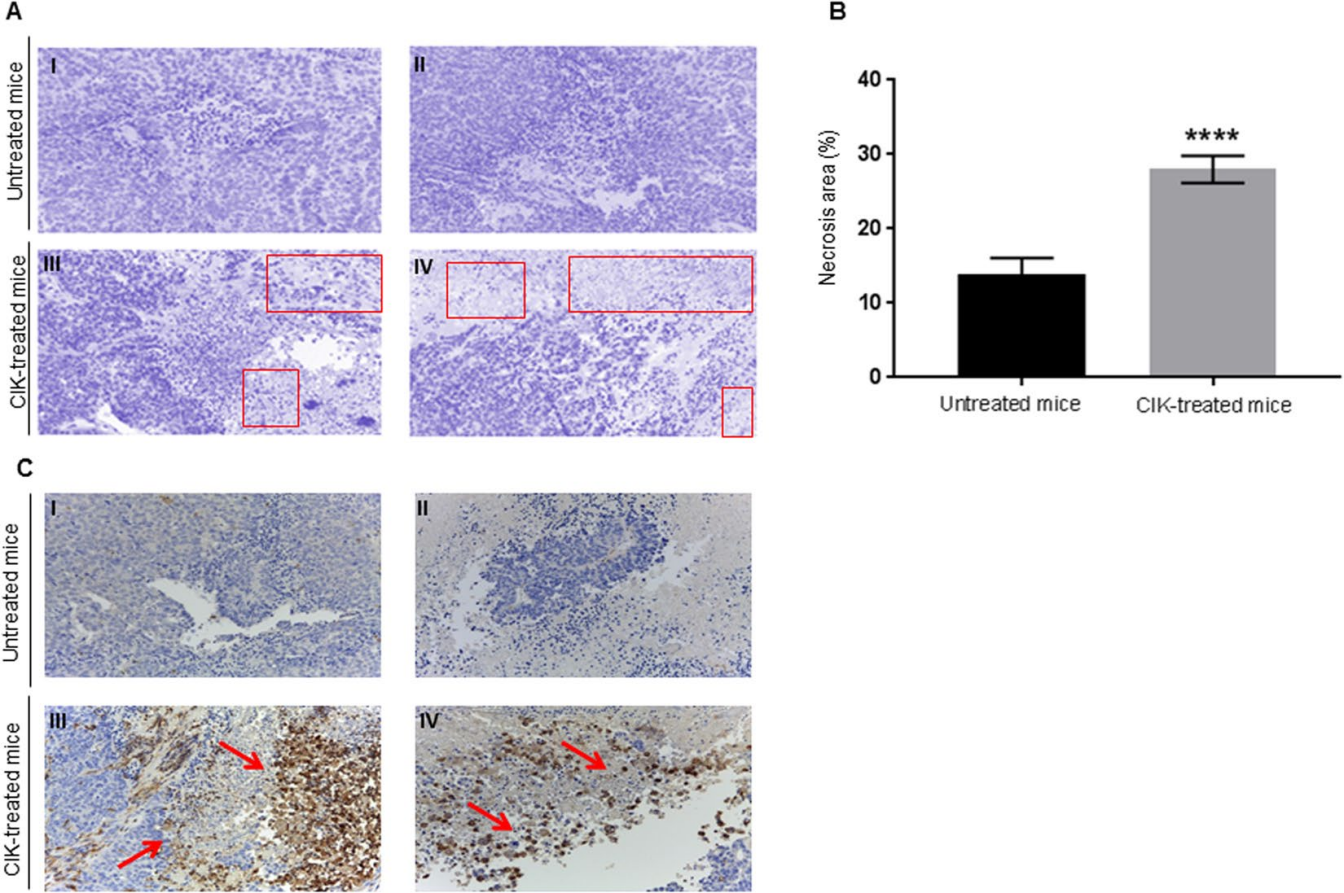
Tumor necrosis and infiltration by CIK *in vivo*. **(A,B)** The infusion of CIK lymphocytes determined higher rates of tumor necrosis compared to untreated controls. Representative images (squares indicate necrotic areas) and cumulative data reported (evaluation by color deconvolution method). (**C)** Representative images (human CD3 IHC staining, Magnification 10×) of CIK tumor infiltration *in vivo*.

The antitumor activity of CIK was explored *in vivo* within a patient-derived high grade serous ovarian cancer xenograft model (PDX) obtained from an omental node of a chemo-naïve 75 years old patient^54^. ϒ null-NOD/SCID mice were subcutaneously injected with the PDX ovarian tumor sample (125 mm^3^, see methods). Starting 2 weeks after tumor implantation, mice (n = 6) were treated with 2 weekly intravenous infusions of mature CIK (1 × 10^7^) for 5 weeks; mice injected with PBS alone were used as the untreated control (n = 6). Adoptive immunotherapy with CIK significantly reduced tumor cell viability, evaluated by tumor-glucose uptake *in vivo*, compared to untreated controls (p = 0.0022) (Fig. 5). Mice were sacrificed 10 days after the last CIK infusion observing *i)* higher rates of necrotic areas in treated mice compared with untreated controls (28% vs 14%, p < 0.0001, panel A and B, Fig. 6) and *ii)* detectable rates of human tumor infiltrating CIK (CD3+, 35% ± 5), mostly located near the necrotic tissue in treated tumors (panel C, Fig. 6).

## Discussion

Our study reports the preclinical activity of HLA-independent CIK immunotherapy within patient-derived ovarian cancer models, including platinum resistant targets.

Novel surgical techniques and new drugs such as PARP inhibitors and antiangiogenics significantly improved EOC prognosis. Nevertheless, overall clinical outcome, especially after failure of platinum-based chemotherapy, is still severe and novel therapeutic strategies are largely awaited^3,55–57^. In recent years preclinical studies already suggested the efficacy of CIK against ovarian cancer cell lines *in vitro* and *in vivo*^51^, underscoring the role of NKG2D in tumor recognition and possible synergism with bispecific antibodies^58^.

We confirmed the importance of NDG2D ligands in this setting, reporting their high expression in pdOVC generated from metastatic tumors relapsed after platinum treatment.

It is worth to note however that blocking the NKG2D receptor on CIK reduced but not completely abrogated their killing activity, suggesting the activity of other molecules (e.g. DNAM, LFA-1) partially contributing to the observed antitumor effect. A deeper comprehension of the hierarchy and role of all CIK ligands is warranted as it could help the identification of predictors of response and, as a consequence, subsets of patients that could better benefit from CIK-based immunotherapy.

Early evidence from clinical trials support the feasibility and safety profile of CIK immunotherapy, with initial signs of activity and positive impact on survival outcomes in multiple solid tumors, including ovarian cancer either in advanced^31,34,38,42,59–61^ or even adjuvant or post-chemotherapy maintenance settings^62^.

A specific, clinical relevant, issue for translational researches is exploring the killing activity of CIK against relapsed chemo-resistant EOC targets.

Our preclinical model is mainly developed within this frame, with the intent of addressing the emerging need for reliable translational platforms in clinical perspective. The experiments with autologous tumor samples are important in the effort of recapitulating the intrinsic, and mostly unknown, biologic elements that regulate the interface between immune effectors and cancer targets. Patient-derived tumor targets in our work were obtained from metastatic ascites. This could be an important aspect, representative of a realistic clinical scenarios. Metastases may have indeed important biologic and immunogenic differences compared with the primary tumors and, in the hypothesis of clinical translations, CIK immunotherapy will certainly be tested in patients with advanced metastatic disease.

Circulating CIK precursors were obtained directly from PBMC of patients with high grade serous EOCs. Importantly, we observed an intense, *ex vivo* expansion rates that were not affected by previous or concomitant platinum based treatments. This supports the concept of a procedure that is clinically exploitable, especially in heavily pretreated patients with platinum resistant EOC.

In a clinical perspective, the safe, simple and cost-effective protocol to generate CIK is a valuable issue; this makes CIK strategy compare favorably with other approaches currently under investigation, that include extensive lymphocyte manipulation or genetic engineering. CIK precursors for clinical use may be obtained by leukapheresis or even limited peripheral blood withdrawals, easily repeatable for patients with lower CIK expansion rates that might undergo multiple CIK *ex vivo* expansion processes. In preparation to clinical trials, the protocol to generate CIK has been recently validated by our and other groups in GMP conditions^63^.

The activity of patient-derived CIK was also confirmed *in vivo* within an ovarian cancer xenograft model^54^.

We performed multiple CIK infusions, hypothesizing that a similar schedule might be replicated also in a hypothetic clinical study. In a clinical prospective, considering the very favorable safety profile of CIK immunotherapy, multiple infusions could be pursued to provide a stronger effect. This animal model, however, was based on a PDX generated from a surgical biopsy collected before the chemotherapy treatment. A more realistic simulation of post-platinum treatment was instead simulated *in vitro*, observing the effective activity of patient-derived CIK versus pdOVC targets that survived a treatment with therapeutic doses (IC_50_) with carboplatin. Of note, the stress-inducible NKG2D ligands trended to increase after platinum treatment, supporting the observed CIK cytotoxicity and providing rational to explore combinatorial/sequential approaches with chemotherapy. In conclusion, in the continuously evolving landscape of EOC, where targeted therapies such as PARP inhibitors are acquiring increasing relevance, there is still room for immunotherapy in multiple clinical settings. However, with the exceptions of limited subsets of tumor histotypes (i.e. clear cell OC), checkpoint inhibitors have not replicated the exciting results obtained in other cancers, framing the rationale to explore alternative or complementary strategies like adoptive immunotherapy.

Probably, considering general immunologic axioms suggesting that low tumor burden may elicit a better immunologic control, the optimal clinical setting to develop CIK immunotherapy is probably that of early disease (e.g. after primary or interval cytoreduction) or non-bulky disease relapse.

CIK activity is not impaired by a restrained neoantigen load, as it seems to be the case in most ovarian cancers, nor by intrinsic defects in antigen presentation, HLA integrity or interferon pathways all reported factors linked to failure of checkpoint inhibitors. Furthermore, CIK are ultimately activated T lymphocytes presenting variable expression of PD-1 and other checkpoints. It is conceivable, and currently under investigation in other malignancies, that their antitumor activity may synergize with checkpoint modulators antibodies.

## Materials and Methods

### Generation and *ex-vivo* expansion of CIK

Peripheral blood mononuclear cells (PBMC) were obtained, by density gradient centrifugation with Lymphoprep (Sentinel Diagnostic), from patients affected by EOC diagnosed at advanced stage at Candiolo Cancer Institute, Fondazione del Piemonte per l’Oncologia (FPO)–IRCCS (Candiolo, Torino, Italy). All individuals provided their written informed consent.

PBMCs were cultured overnight in cell culture flasks at a cell density of 1.5 × 10^6^/mL RPMI (Gibco BRL) supplemented with 10% FBS (Sigma) and IFN-γ (1.000 U/ml, PeproTech). After 24 hours in culture anti-CD3 antibody (OKT3, 50 ng/mL, PharMingen) and recombinant human IL-2 (300 U/mL, Miltenyi Biotec S.r.l.) were added. Fresh medium with IL-2 was added as needed.

Phenotype of CIK was analyzed by standard flow cytometric assays. The following monoclonal antibodies (mAb) were used: CD3 (Anti-Human CD3 FITC Mouse, BD PHarmingen™), CD4 (Anti-Human CD4 PE, MACS MiltenyiBiotec), CD8 (Anti-Human CD8 PE, MACS MiltenyiBiotec), CD56 (Anti-Human CD56 APC, MACS MiltenyiBiotec) and CD314–APC (anti-NKG2D; MACS MiltenyiBiotec).

### Establishment of patient derived ovarian cancer cell lines (pdOVC)

Freshly isolated ascites were obtained in a sterile vacuum bottle; platinum resistant patients provided consent under institutional review board–approved protocols. 25 ml of fresh ascites fluids were transfer in vented caps T-75cm^2^ tissue cultures flasks (Corning/Costar) with 25 ml MCDB131/DMEM High-Glucose (1:1) with the addition of penicillin (50 U/mL), streptomycin (50 μg/mL), Glutamax 100 × (all from Gibco BRL), 10% heat-inactivated FBS (Euroclone).

After 3–4 days medium was completely changed; when cells achieved confluence were washed with PBS and trypsinized using trypsin-EDTA solution for 5 minutes at 37 °C.

To confirm that our pdOVC had preserved the morphology and cellular details of tissue samples were formalin fixed and paraffin embedded; the samples were analyzed in collaboration with Pathology department (FPO–IRCCS, Candiolo, Torino, Italy) to confirm the expression of the ovarian cancer markers Wilms’s Tumor 1 (WT1) and epithelial marker cytokeratin 7 (CK7).

Phenotypic analysis of pdOVC was performed using the following fluorescein isothiocyanate (FITC), phycoerythrin (PE), allophycocyanin (APC), (Thermo Fisher Scientific) BV421 Violet1, PerCP/CY5, PE-vio770, PE vio615, FITC VioBright, -conjugated mouse mAbs against HLA-ABC (anti-HLA-ABC-FITC, BD Pharmingen) and CIK target molecules (anti-MICA/B, BD Pharmingen; anti-ULBPs, R&D System, Space Import Export; PD1 Miltenyi Biotec; TIM3 Miltenyi Biotec; LAG3 Miltenyi Biotec; TIGIT eBioscience, Inc;, DNAM BD Pharmingen;,NKp44 Miltenyi Biotec; NKp30 Miltenyi Biotec; NKp46 Miltenyi Biotec; TCRαβ, Caltag Laboratories Burlingame ca; TCRϒδ BD Pharmingen; 62 L Miltenyi Biotec; CD45RA Miltenyi Biotec.

We also tested commercially available ovarian cancer cell lines: OVCAR-3, OVCAR-5, IGROW-1, A2780 e OAW42 obtained from American Type Culture Collection (ATCC). All cell lines have been characterized by the provider and maintained as suggested. Phenotypical analysis was conducted as previously described.

### *In vitro* assays of cytotoxicity of patient-derived CIK

The tumor-killing ability of patient-derived CIK was assessed *in vitro* against ovarian cancer cell lines (either autologous or allogeneic).

The antitumor activity of patient derived CIK was evaluated by a bioluminescent tumor cell viability essay (CellTiter-Glo, Promega). CIK and tumor target cells were co-coltured at various effectors/target ratios (40:1, 20:1, 10:1 5:1, 2,5, 1:1, 1:2, 1:4) for 72 hours in 200 μL of culture medium with 300 U/mL IL2 at 37 °C, 5% CO2 and the measurements were recorded with a GloMax 96 Microplate Luminometer (Promega). Growth inhibition at various effectors/target ratios was normalized to CIK untreated tumor cells.

Whenever possible the antitumor activity of patient derived CIK was additionally confirmed by cytofluorimetric analysis. Target cells were stained in accordance with the manufacturer’s protocol with the vital dye CFSE (5, 6-carboxyfluorescein diacetate succinimidyl ester; Molecular Probes) or PKH26 Red Fluorescent Cell Linker kit (Sigma Aldrich). The immune-mediated killing was determined evaluating cell viability by flow cytometry (Cyan ADP, Dako), after 24 hours incubation with expanded CIK cells, as previously described, according to the formula: experimental − spontaneous mortality/(100 − spontaneous mortality) × 100. Cytotoxicity was calculated through flow cytometry (Cyan ADP, Beckman Coulter s.r.l.) using DAPI permeability assay (Thermo Fisher Scientific) of target cells. As descriptive statistic al analysis, medians and ranges, mean ± SEM were used as appropriate; statistical significance was expressed as true *P* value. All *P* < 0.05 were considered statistically significant. Statistical analysis was conducted using software Graph Pad Prism 8.0.

In blocking experiments CIK were pre-incubated with 40 μg/mL of inhibitory anti-NKG2D neutralizing antibody (Clone #149810, R&D Systems).

### *In vitro* assessment of pdOVC sensitivity to carboplatin

pdOVC cells were seeded into 96-well plates (3, 5–4 × 10^4^ cells/well). After overnight incubation, cells were treated with the half-maximal inhibitory concentration (IC_50_) dose carboplatin Carboplatin (Pfizer, 450 mg/45 ml Injection) for 72 h. The cell viability was determined with CellTiter-Glo assay.

### *In vivo* antitumor activity of patient-derived CIK

EOC patient derived xenograft (PDX) model of was carried out as previously described^54^. Briefly, we implanted subcutaneously in right flank of severely immunocompromised NOD/Shi-scid/IL-2R γnull mice PDX line tumor samples of 125 mm^3^.Tumor size was evaluated twice-weekly with digital caliper and volume was calculated using the formula 4/3π*(d/2)^2^*D/2, where d is the minor tumor axis and D is the major tumor axis. All animal procedures were approved by the institutional competent Committee.

Starting 2 weeks after implantation, 6 mice of the “treated group” (CIK) received, twice a week for 5 weeks, intravenous infusions of 1 × 10^7^ mature autologous CIK, resuspended in 1 × PBS (200 μL total volume injected), while 6 mice of the “control group” (CTRL) received PBS alone.

The analysis of tumor metabolic activity was performed using Fluorescent probe XenoLightRediJect 2-DeoxyGlucosone (DG)-750 (Caliper Life Sciences, USA). Fluorescent probe was injected through the mouse tail vein. The tumor location and glucose accumulation was acquired by *IVIS*® SpectrumCT (Perkin Elmer, Waltham, MA, USA) 4 hours after injection of 2-DG750 and analyzed using Living Image Software (Perkin Elmer, Caliper Life Sciences). To calculate metabolic activity of tumor mass, we acquired 3D images and the ratio between total fluorescence emitted and total number of tumor mass voxels, was calculated. Also in this case, we represented the mean value ± SEM. Statistical analysis was performed by using T software Graph Pad Prism 8. The evaluation of necrotic versus alive cells area was conduct considering the color deconvolution method (acquisition of 10 field for each sample, 10X of magnitude).

Eosin/hematoxylin-stained cells were digitally separated using ImageJ software (version 1.46c; WS Rasband, National Institutes of Health, Bethesda, MD, USA,) and an ImageJ plugin for color deconvolution^64^. The hematoxylin deconvoluted image was subjected to histogram analysis to identify nuclei of alive cells. The total area of alive nuclei were subtracted from estimated total field area (measured and calculated as average in 4 field fully covered of alive cells and used ad reference data) and translate in percentage. Significant Statistical analysis was conducted using software Graph Pad Prism 8.The collection of clinical samples was undertaken with the understanding and written consent of each subject according to the PROFILING Protocol, conformed to the standards set by the Declaration of Helsinki and approved by the Regione Piemonte Ethical Committee (approval no 5141 on 9/3/2011) and then by the IRCCS Ethical Committee (approval no. 192/2016 on 19/7/2016).

All animal procedures were approved by the local Ethical Commission and by the Italian Ministry of Health in accordance with EU Directive 2010/63/EU for animal experiments**;** first authorization was sent on 12/7/2012 and, following subsequent regulations, approved on 14/01/2016 (no. 16/2016-PR) and extended for two additional years on 17/9/2018.

### Immunohistochemistry

5 μm in thickness FFPE tissue sections were utilized to visualize morphology of tumor tissues via H&E staining and to detect human lymphocytes with CD3 antibody (DAKO, Carpinteria, CA), for CD3 staining, deparaffinization, rehydration and target retrieval were performed following Dako instructions. To quantify data, at least 10 images of each sample were acquired by optical microscope (20×) connected with charge-coupled device (CCD) camera and analyzed by using automatic counter software (NIH ImageJ, W. Rasband, NIH) software. The percentage of necrosis was estimated calculating the difference between a “standard control area” covered by healthy cells and the treated and untreated groups. To define “standard control area” we considered 5H&E fields in which all the area was occupied by round and clearly healthy cells. To calculate the percentage of human lymphocytes in tumors, we compared in the number of all nuclei present in the field versus the number of CD3 positive cells (which are completely absent in non treated tumors).

## Supplementary information

Supplementary information.
