# Supplementary material for: Preclinical immunotherapy with Cytokine-Induced Killer lymphocytes against epithelial ovarian cancer
Source: Sci Rep. 2020 Apr 15;10:6478. doi: 10.1038/s41598-020-63634-z (PMC7160190; doi:10.1038/s41598-020-63634-z)
Supplement: Supplementary file 1 — Supplementary information. [file 41598_2020_63634_MOESM1_ESM.pdf]

Preclinical immunotherapy with Cytokine-Induced Killer lymphocytes against epithelial ovarian cancer

Capellero S[1,2], Erriquez J[2], Melano C[1,2], Mesiano G[1,2], Genta S[1,2], Pisacane A[2], Mittica G[3], Ghisoni E[1,2], Olivero M[1,2], Di Renzo MF[1,2], Aglietta M[1,2],Sangiolo D[1,2*],Valabrega G[1,2°*]

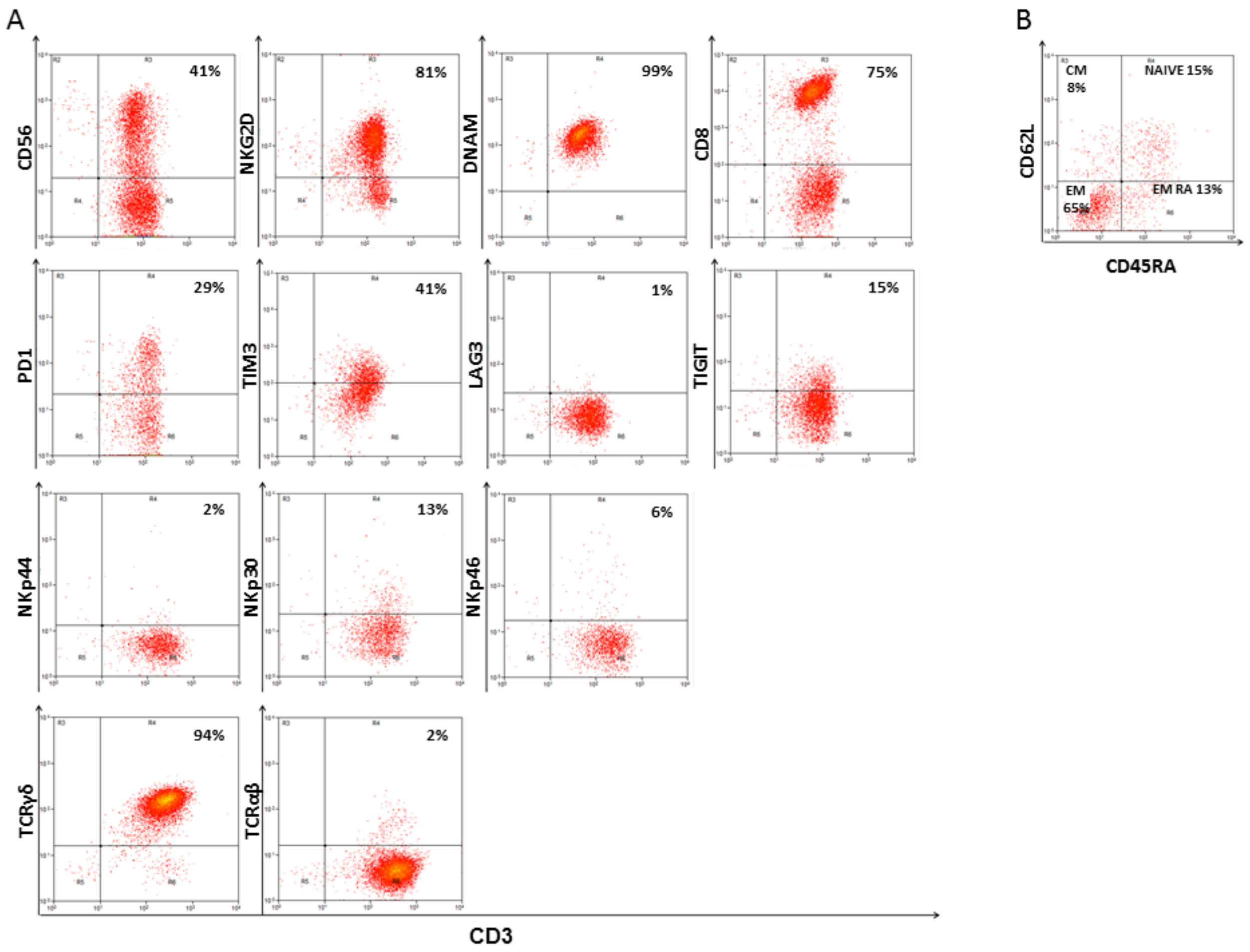

Supplementary 1: **Phenotype of mature CIK**.

A,B) The phenotype of mature CIK lymphocytes was analyzed by standard flow cytometric assays. Representative dot plots, including the main activation markers, Immune-checkpoints and functional subsets are reported.

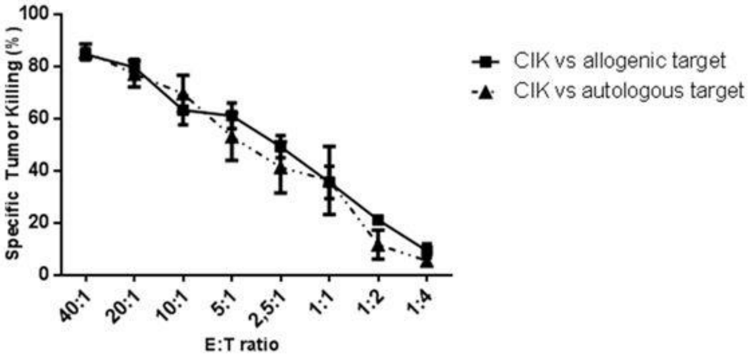

Supplementary 2: **Tumor Killing activity of autologous and allogeneic CIK lymphocytes.** The intensity of CIK tumor killing observed within autologous settings (n=6) was comparable to that observed with allogeneic CIK (n=7). Tumor killing was evaluated by CellTiter-Glo Luminescent Cell Viability Assay. Mean values of tumor-specific killing are reported at decreasing CIK/target ratios

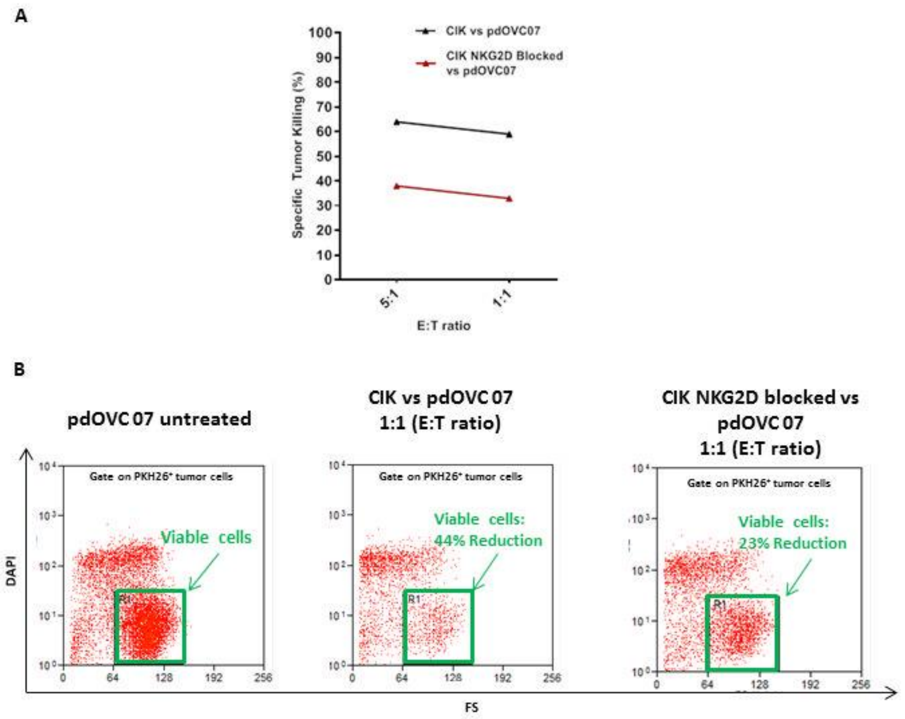

Supplementary 3: **Blocking of NKG2D receptor reduces CIK tumor killing activity**. Blocking of NKG2D receptor with neutralizing monoclonal antibody resulted in reduction of tumor killing activity by CIK lymphocytes. **A)** Quantification of cytotoxicity reduction by NKG2D blocking and **B)** representative flow-cytometry plots of the tumor killing essay are reported.
